# Multiparametric magnetic resonance imaging for quantitation of liver disease: a two-centre cross-sectional observational study

**DOI:** 10.1038/s41598-018-27560-5

**Published:** 2018-06-15

**Authors:** Natasha McDonald, Peter J. Eddowes, James Hodson, Scott I. K. Semple, Nigel P. Davies, Catherine J. Kelly, Stella Kin, Miranda Phillips, Amy H. Herlihy, Timothy J. Kendall, Rachel M. Brown, Desley A. H. Neil, Stefan G. Hübscher, Gideon M. Hirschfield, Jonathan A. Fallowfield

**Affiliations:** 1 MRC/University of Edinburgh Centre for Inflammation Research, Queen’s Medical Research Institute, Edinburgh, EH16 4TJ UK; 2 Centre for Liver Research and NIHR Birmingham BRC, University of Birmingham, Birmingham, B15 2TT UK; 3 NIHR Nottingham BRC, Nottingham University Hospitals NHS Trust and the University of Nottingham, Nottingham, NG1 5DU UK; 4 Institute of Translational Medicine, University Hospitals Birmingham NHS Foundation Trust, Birmingham, B15 2TT UK; 5 BHF/University of Edinburgh Centre for Cardiovascular Science, Queen’s Medical Research Institute, Edinburgh, EH16 4TJ UK; 6 Imaging and Medical Physics, University Hospitals Birmingham NHS Foundation Trust, Birmingham, B15 2TH UK; 7 Perspectum Diagnostics Ltd., Oxford Centre for Innovation, Oxford, OX1 1BY UK; 8 Division of Pathology, Royal Infirmary of Edinburgh, Edinburgh, EH16 4SA UK; 9 Department of Cellular Pathology, Queen Elizabeth Hospital, Birmingham, B15 2TH UK

## Abstract

Liver*MultiScan* is an emerging diagnostic tool using multiparametric MRI to quantify liver disease. In a two-centre prospective validation study, 161 consecutive adult patients who had clinically-indicated liver biopsies underwent contemporaneous non-contrast multiparametric MRI at 3.0 tesla (proton density fat fraction (PDFF), T1 and T2* mapping), transient elastography (TE) and Enhanced Liver Fibrosis (ELF) test. Non-invasive liver tests were correlated with gold standard histothological measures. Reproducibility of Liver*MultiScan* was investigated in 22 healthy volunteers. Iron-corrected T1 (cT1), TE, and ELF demonstrated a positive correlation with hepatic collagen proportionate area (all *p* < 0·001). TE was superior to ELF and cT1 for predicting fibrosis stage. cT1 maintained good predictive accuracy for diagnosing significant fibrosis in cases with indeterminate ELF, but not for cases with indeterminate TE values. PDFF had high predictive accuracy for individual steatosis grades, with AUROCs ranging from 0.90–0.94. T2* mapping diagnosed iron accumulation with AUROC of 0.79 (95% CI: 0.67–0.92) and negative predictive value of 96%. Liver*MultiScan* showed excellent test/re-test reliability (coefficients of variation ranging from 1.4% to 2.8% for cT1). Overall failure rates for Liver*MultiScan*, ELF and TE were 4.3%, 1.9% and 15%, respectively. Liver*MultiScan* is an emerging point-of-care diagnostic tool that is comparable with the established non-invasive tests for assessment of liver fibrosis, whilst at the same time offering a superior technical success rate and contemporaneous measurement of liver steatosis and iron accumulation.

## Introduction

With the increasing prevalence of chronic liver disease (CLD), particularly related to the global epidemic of non-alcoholic fatty liver disease (NAFLD), there is a pressing need for reliable and widely applicable methods to diagnose, stratify and monitor liver disease progression/regression that are acceptable to patients and cost-effective for healthcare providers^1^. Three independent reports have highlighted the need for the early detection of liver disease, including the UK Chief Medical Officer report (2011)^2^, the All-Party Parliamentary Hepatology Group Inquiry^3^ and the Lancet Commission in 2014^4^. The early detection of liver disease is important, as effective intervention can prevent progression to cirrhosis and hepatocellular carcinoma and thereby reduce the economic burden of liver disease and save lives^5^. Despite this, existing diagnostic pathways for detection and onward referral of suspected CLD in primary care are based on traditional liver enzyme tests, which lack accuracy and contribute to late diagnosis, whilst staging of liver disease in secondary care and evaluation of drug efficacy in clinical trials remains anchored to the liver biopsy. Liver biopsy is commonly used to assess liver disease but has drawbacks, including sampling variability (especially in liver conditions where histological changes are patchy), interobserver disagreement and a potential risk of complications, including patient discomfort and anxiety^6^. A lack of robust, validated non-invasive tests has hindered drug development efforts for CLD; suitable alternative methods to biopsy could enable trial enrichment and/or read out as an early efficacy signal or surrogate endpoint.

Transient elastography (TE) (FibroScan; Echosens, Paris, France) is an established non-invasive test with good diagnostic accuracy for ruling out advanced fibrosis or cirrhosis (≥Metavir stage F3) and emerging prognostic capability^7^, but there is an average failure rate of 18·4%^8^ and cut-off values of liver stiffness for the different stages of liver fibrosis are not well established^9^. Moreover, both operator-related and patient-related factors produce significant variations in liver stiffness measurements, limiting its potential use for monitoring disease progression^10^.

A range of serological tests are available for the assessment of liver fibrosis. These include simple marker panels based on routine blood tests with or without clinical parameters (e.g. AST to platelet ratio index (APRI) and Fibrosis-4 (FIB-4) test) and more complex fibrosis-orientated marker panels (e.g. Enhanced Liver Fibrosis (ELF; iQur Ltd., London, UK) test and FibroTest (BioPredictive, France) which are analysed using patented algorithms. Although these serological tests have been validated in many chronic liver diseases and may predict clinical outcomes^11,12^, they fail to classify a significant proportion of patients who fall into the ‘grey zone’ of indeterminate values.

Multiparametric MRI techniques have shown promise for the quantitative assessment of many chronic conditions in the heart, breast, prostate and musculoskeletal system, obviating the need for invasive tissue characterisation in many patients^13^. In the liver, MRI proton density fat fraction (PDFF) has been shown to be highly accurate and reproducible for the detection and quantification of hepatic steatosis, independent of field strength, and can detect changes in hepatic fat as small as 1%^14,15^. Magnetic resonance elastography (MRE) is a phase-contrast MRI technique that measures liver stiffness as a surrogate of fibrosis. MRE has high accuracy for the diagnosis of significant liver fibrosis and cirrhosis, but it is not yet known whether it is sufficiently sensitive or dynamic for the longitudinal monitoring of fibrosis progression/regression^16^. Liver*MultiScan* (LMS) (Perspectum Diagnostics Ltd., Oxford, UK) is a proprietary, CE-marked, Food and Drug Administration (FDA) 510(k) cleared multiparametric MRI methodology encompassing measurement of hepatic fibro-inflammatory injury, fat and iron^17^. A derived Liver Inflammation and Fibrosis (LIF) score has been shown in a single-centre CLD cohort to correlate with histological fibrosis staging, and a LIF score <2 had a NPV of 100% for a clinical outcome over a median follow up of 27 months^18^. We conducted a larger, independent, two-centre prospective validation study in an unselected secondary care population, with the primary objective of evaluating the ability of LMS to accurately measure hepatic inflammation and fibrosis, fat and iron compared to liver biopsy as the reference standard. The secondary objectives were to assess the performance of LMS in detecting clinically significant liver disease, to compare its diagnostic performance to TE and ELF, and to determine the reproducibility and repeatability of the technique.

## Methods

### Study design and population

This was a prospective two-centre validation study that represented level 1b evidence for diagnostic test assessment^19^ and reported to the Standards for Reporting Diagnostic Accuracy^20^. 161 unselected consecutive adult patients booked for a standard-of-care liver biopsy to investigate known or suspected liver disease, including patients post liver transplantation, were included. Data collection took place at two large tertiary UK liver centres (Queen Elizabeth Hospital Birmingham and Royal Infirmary of Edinburgh) between February 2014 and September 2015. Patient exclusion criteria were inability or unwillingness to give fully informed consent, any contraindication to MRI, and liver biopsy targeted at a focal liver lesion. Participants underwent contemporaneous multiparametric MRI (LMS), TE and analysis of blood biomarkers including the ELF test, followed by liver biopsy performed within 2 weeks of non-invasive assessments. Reference MRI data were also collected from 22 male and female adult healthy volunteers with no known liver disease and body mass index (BMI) <25 kg/m^2^. All study investigations were performed in a fasted state (minimum of four hours).

The study was conducted in accordance with the ethical principles of the Declaration of Helsinki 2013 and Good Clinical Practice guidelines. It was approved by the institutional research departments and the National Research Ethics Service (14/WM/0010). The study was registered with the ISRCTN registry (ISCRTN39463479) and the National Institute for Health Research portfolio (15912). All patients and volunteers gave written informed consent.

### Histological analysis of liver biopsy samples

All biopsies were reported by four independent expert liver histopathologists, and adequacy assessed using the definition of the Royal College of Pathologists^21^. All biopsies were staged for fibrosis using modified Ishak score (MIS) (scale 0–6; Supplementary Table 1) and collagen proportionate area (CPA) was calculated by morphometry after Picrosirius red staining, as previously described^22^. Liver inflammation was graded independently (none/minimal, mild, and moderate/severe) from histopathology reports by two assessors (NM, PJE) blinded to patient characteristics and non-invasive assessment data. Discordance was adjudicated by a third blinded observer (TJK). Liver fat was graded 0–3 based on the percentage of hepatocytes in the biopsy containing a fat globule: 0 (<5%); 1 (5–32%), 2 (33–66%), and 3 (>66%)^23^. Liver iron was detected using Perls’ stain and semi-quantified using a five-tier grading system (0: no iron deposition to 4: severe iron deposition)^24^. As there is considerable interobserver variation in liver biopsy reporting, 45 randomly-selected biopsies were independently re-scored by two liver histopathologists (SH, TJK) blinded to the clinical data and previous pathology reports, to generate five observer pairs.

### Magnetic resonance imaging and image analysis

Non-contrast MR acquisitions were performed with the patient in the supine position using a 3.0 tesla Siemens Verio MRI scanner system (Siemens Healthcare GMBH, Erlangen, Germany). MRI operators and image data assessors were blinded to the indication for liver biopsy and to the patients’ clinical details. MRI acquisition protocols (transverse abdominal T1 and T2* maps, proton density fat fraction (PDFF, %)) and image analysis were performed as previously described^17^, but with a shorter acquisition time of approximately 10 minutes. Great care was taken to optimise the acquisition protocol to minimise the impact of breathing motion and other artefacts. This included extensive training of the MR technicians on healthy volunteers and strict quality control throughout the process. Full details of the MR protocol and reproducibility and repeatability studies are provided in Supplementary Methods.

### Blood analysis

All patients had full blood count, coagulation profile, serum biochemistry and ELF test measured prior to liver biopsy.

### Transient elastography

One-dimensional TE was performed using FibroScan by fully trained and certified operators, using either an M or XL probe for obese subjects to obtain ten valid readings, with a success rate of at least 60% and IQR <30% of the median result. XL probe was used in 81 patients (50% of study cohort). Controlled attenuation parameter (CAP) estimation was unavailable at the Royal Infirmary of Edinburgh.

### Sample size calculation

Based on data from a previous study, the distribution of patients across the 4 groups (Ishak 0, 1–2, 3–4 and 5–6) was 9%: 52%: 17%: 22%. The pooled value for the difference in cT1 between sequential groups in this data were found to be approximately 90 ms. Due to the large differences in the standard deviations across the groups, the study was powered on the “worst case” pairwise comparison, based on the combination of the observed standard deviation and the proportional sample size. This was between Ishak 3,4 (SD = 57, 17% of patients) and Ishak 5,6 (SD = 90, 22% of patients).

For a comparison between these groups using an alpha level of 0.8% (i.e. 5% after adjustment for 6 comparisons), sample sizes of 12 and 22 for Ishak stages 3,4 and 5,6 respectively would be sufficient to detect a difference of 90 ms in cT1 at 80% power. Assuming that the distribution of cases was similar to the previous study, this meant that a sample size of 100 patients (9, 52, 17 and 22 in the four groups) would be sufficient to detect a difference between groups of 90 ms at 80% power and with 5% alpha. We targeted a total recruitment number of 150 to account for the ~10% of biopsies that yield inadequate samples for analysis and participant non-attendance.

### Statistical analysis

Repeatability of the MRI data was assessed using Bland-Altman (B-A) plots, 95% limits of agreement, Pearson correlation coefficients, and paired *t*-tests, to assess the level of bias. The mean coefficient of variation (CoV) was also calculated, as the average of the CoV for each patient. Interobserver agreement for biopsies was assessed using quadratic weighted Kappa^25^. Associations between continuous and ordinal variables were assessed using the Jonckheere-Terpstra test of trend, followed by pairwise post-hoc tests, where significant. For comparisons between two groups, Mann-Whitney tests were used, whilst associations between two continuous variables were assessed using Spearman’s correlation coefficient (r_s_). Multivariable analysis was performed to assess the relationship between fibrosis and the diagnostic tests, after accounting for the effect of inflammation, using two-way ANOVA with inflammation, fibrosis and an interaction term as factors. TE values were log-transformed, prior to analysis, to improve model fit.

Diagnostic performance of tests was assessed using receiver operating characteristic (ROC) curve analyses. Where applicable, the areas under the ROC curves (AUROCs) were compared using the “roccomp” command in Stata 14 (StataCorp LP., College Station, TX), with significance tests followed by Bonferroni-adjusted post-hoc pairwise comparisons. Net reclassification index (NRI) was calculated as a percentage difference between cases correctly classified by the reference and alternative tests. Statistical analysis was performed using IBM SPSS Statistics for Windows version 22 (IBM Corp, Armonk, NY) and GraphPad Prism version 6.0 (GraphPad Software, USA). Variables were summarised as means (±standard deviation (SD)) if normally distributed and as medians with interquartile range (IQR) if not. A *p*-value of less than 0·05 was considered statistically significant.

### Data availability

The datasets generated during and/or analysed during the current study are available from the corresponding author on reasonable request.

## Results

**Figure 1:**
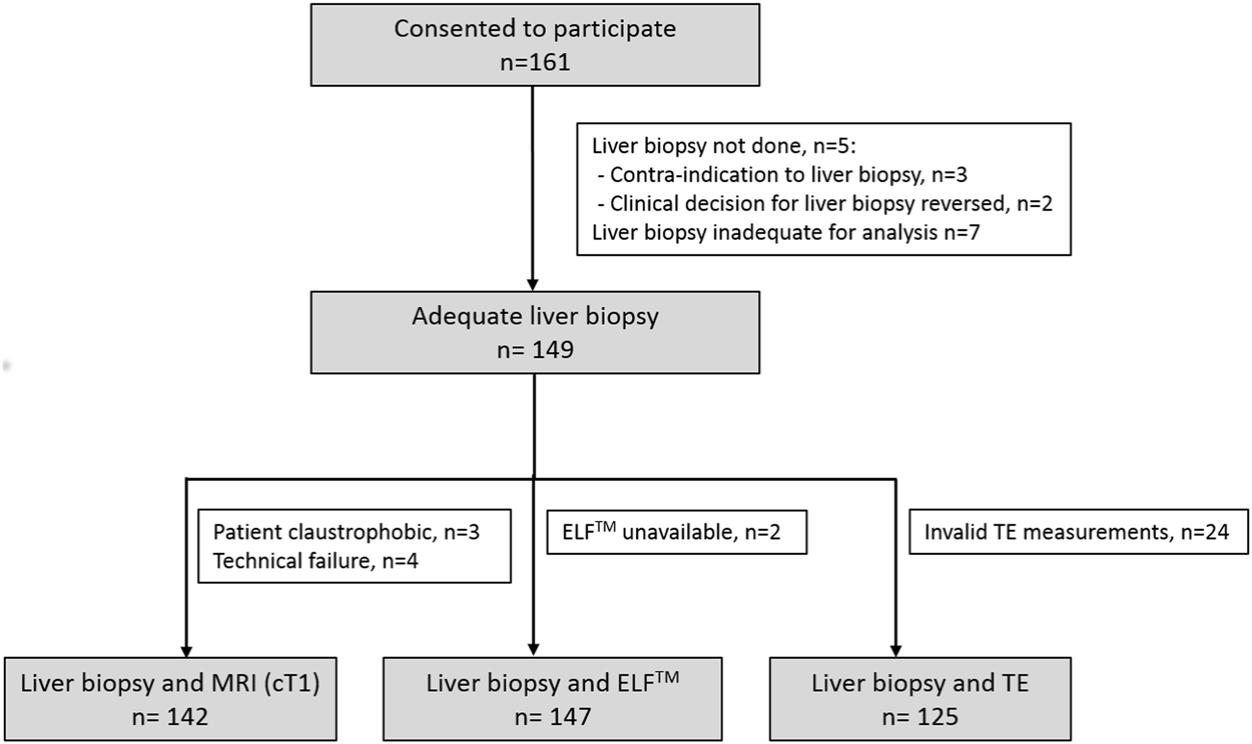
Study flowchart.

A total of 161 patients consented to participate and 156 were biopsied (Fig. 1). Seven biopsies (4·5%) were considered inadequate for interpretation and excluded from further analysis. Median biopsy length after processing was 25 mm (IQR 22–28). Interobserver agreement for histological assessment of liver fat, fibrosis and iron for the 45 samples that were reassessed was 71% (kappa = 0·81, almost perfect), 38% (kappa = 0·66, substantial), and 82% (kappa = 0·24, fair), respectively (Supplementary Table 2). The kappa statistic for iron was low, relative to the percentage agreement, due to the fact that most patients were in the ‘no iron deposition’ group, resulting in low inter-case variability^26^.

Baseline demographic and clinical characteristics of study participants with adequate biopsies are summarised in Table 1.

**Table 1:** Baseline demographic and clinical characteristics of the 149 study participants with adequate biopsy data.

	Statistic
Gender (% Male)	89 (60%)
Age (years)	50 (±13)
Liver transplant	34 (23%)
**Anthropometric data**	
Weight (kg)	85 (±19)
BMI (kg/m^2^)	29·7 (±6·7)
Waist:Hip Ratio	
*Male*	0·95 (±0·08)
*Female*	0·86 (±0·09)
**Liver Enzymes**	
Bilirubin (IU/L)	13 (8–18)
Alanine aminotransferase (IU/L)	58 (34–110)
Aspartate aminotransferase (IU/L)	47 (30–80)
Gamma-glutamyl transpeptidase (IU/L)	103 (52–244)
Alkaline phosphatase (IU/L)	103 (78–165)
**Fibrosis stage** (modified Ishak score)	
*0*	30 (20%)
*1*	27 (18%)
*2*	24 (16%)
*3*	33 (22%)
*4*	9 (6%)
*5*	7 (5%)
*6*	18 (12%)
**Final diagnosis post biopsy**	
*NAFLD*	53 (36%)
*Autoimmune liver disease*	25 (17%)*
*Viral hepatitis*	20 (13%)**
*Normal*	12 (8%)
*Other*	39 (26%)

Data reported as mean (±SD), median (IQR), or n (%), as applicable. *13 AIH, 3 PBC, 3 PSC, 6 overlap syndromes and autoimmune cholangiopathies. **13 patients post-transplant.

### Repeatability and reproducibility of non-invasive tests

Multiparametric MRI was well-tolerated, with a claustrophobia rate of only 1·9% (n = 3). Technical failure of the MRI scanner (shut-down and data loss) affected 2·5% (n = 4) of patients, the remaining 96% (n = 154) all had valid cT1 and T2* data for assessment of fibro-inflammatory injury and liver iron, regardless of BMI. PDFF data were not acquired in the first 48 patients (31%). After updating the protocol, the subsequent 106 (69%) patients all had valid liver fat measurements. The MRI repeatability and reproducibility data are summarised in Table 2, showing significant inter-scan and inter-subject agreement and no evidence of a post-prandial effect.

**Table 2:** Repeatability of multiparametric MRI.

	cT1	T2*	PDFF
**Scan-Rescan**			
Mean CoV (%)	2·1	2·6	8·8
Pearson’s r (95% CI)	0·73 (0·19–0·93)	0·99 (0·97–1·0)	0·99 (0·96 to 1·0)
Bland-Altman Analysis^‡^			
*Bias (mean (*±*SD))*	−13 (35)	−0·05 (0·83)	−1·1 (2·3)
*p-value* ^†^	0·285	0·862	0·250
*95% limits of agreement*	−81 to 56	−1·7 to 1·6	−5·7 to 3·5
**10 week time course**			
Mean CoV (%)	2·8	6·6	NA^#^
**Fasted-Fed**			
Mean CoV (%)	1·40	7·4	NA^##^
Pearson’s r (95% CI)	0·94 (0·82–0·98)	0·90 (0·71–0·97)	
Bland-Altman Analysis			
*Bias (mean*±*SD)*	−3 (22)	1·4 (2·9)	
*p-value*^*†*^	0·606	0·070	
*95% limits of agreement*	−46 to 40	−4·2 to 7·1	

^#^All healthy volunteers had PDFF of < 2%; ^##^only 2 healthy volunteers had PDFF > 2%. ^†^From a paired *t*-test^,^ to test for significant bias. ^‡^See Supplementary Fig. 1 for B-A plots.

Subsequent to this study, we have performed an independent clinical evaluation of the test-retest performance of LMS using both healthy controls and patient volunteers (n = 46) (unpublished data). Consistent with the results reported in this manuscript, the 95% limits of agreement for the mixed healthy control/patient population was −60.5 ms to 49.5 ms with a bias (±SD) of −5.5 ms (±28.1 ms) and a mean CoV of 2.0%. The B-A plot showed no evidence that the test-retest performance is different in patients vs. controls.

The TE failure rate was 15% (n = 24) overall, 7·7% for patients with BMI < 30 and 25% for patients with BMI ≥ 30. One patient was unsuitable for TE due to the presence of ascites detected by MRI. Three patients (1·9%) had unavailable ELF data.

### Assessment of hepatic fibro-inflammatory injury by multiparametric MRI, TE and ELF test

cT1 (r_s_ = 0·33), ELF (r_s_ = 0·41) and TE (r_s_ = 0·52) were all positively associated with liver fibrosis as assessed by CPA (all *p* < 0.001). ROC curve analysis using only valid measurements (Table 3) found no significant difference between the accuracy of the three tests for identifying patients with any (MIS ≥ 1) fibrosis (*p* = 0·085). However, TE was superior to both cT1 and ELF for identification of patients with moderate-severe (MIS ≥ 3) fibrosis (*p* = 0·022, 0·005), and severe (MIS ≥ 5) fibrosis (*p* = 0·003, < 0·001). Following exclusion of post-liver transplant patients, TE retained superiority for identification of only severe (MIS ≥ 5) fibrosis (*p* = 0.002, 0.029) (Supplementary Table 3).

**Table 3:** Diagnostic accuracy of multiparametric MRI, ELF and TE in detecting any, moderate-to-severe and severe liver fibrosis.

Fibrosis stage (MIS)	Multiparametric MRI (n = 142)							ELF test (n = 147)						TE (n = 125)	*p*-value*
	AUROC (95% CI)	Cut off levels		Se	Sp	PPV	NPV	AUROC (95% CI)	Cut off levels	Se	Sp	PPV	NPV	AUROC (95% CI)	
		**cT1 (ms)**	**LIF**												
≥1	0·72 (0·61–0.83)	800	1	86	38	84	41	0·79 (0·71–0·88)	7·7	92	24	83	44	0·83 (0·74–0·92)	0.085
≥3	0·72 (0·63–0·80)	875	2	88	51	60	83	0·70 (0·61–0·78)	9·8	49	77	65	64	0·84 (0·76–0·91)	** < 0.001**
≥5	0·72 (0·64–0·81)	950	3	71	64	28	92	0·68 (0·57–0·79)	11·3	19	91	31	84	0·86 (0·79–0·93)	** < 0.001**

Se, sensitivity; Sp, specificity; NPV, negative predictive value; PPV, positive predictive value. All AUROCs were significant at *p* < 0·001. *p-values com*p*aring the AUROCs of multiparametric MRI vs. ELF vs. TE for the n = 117 patients with data available for each measure. Significant comparisons (bold) were followed by post-hoc pairwise comparisons, the *p*-values of which were Bonferroni corrected to account for multiple comparisons. These comparisons found TE to be significantly superior to both multiparametric MRI and ELF test in the detection of MIS ≥ 3 (*p* = 0.022, 0.005) and MIS ≥ 5 (*p* = 0.003, < 0.001). **TE cut off levels not included as they are specific to aetiology of liver disease.

**Figure 2:**
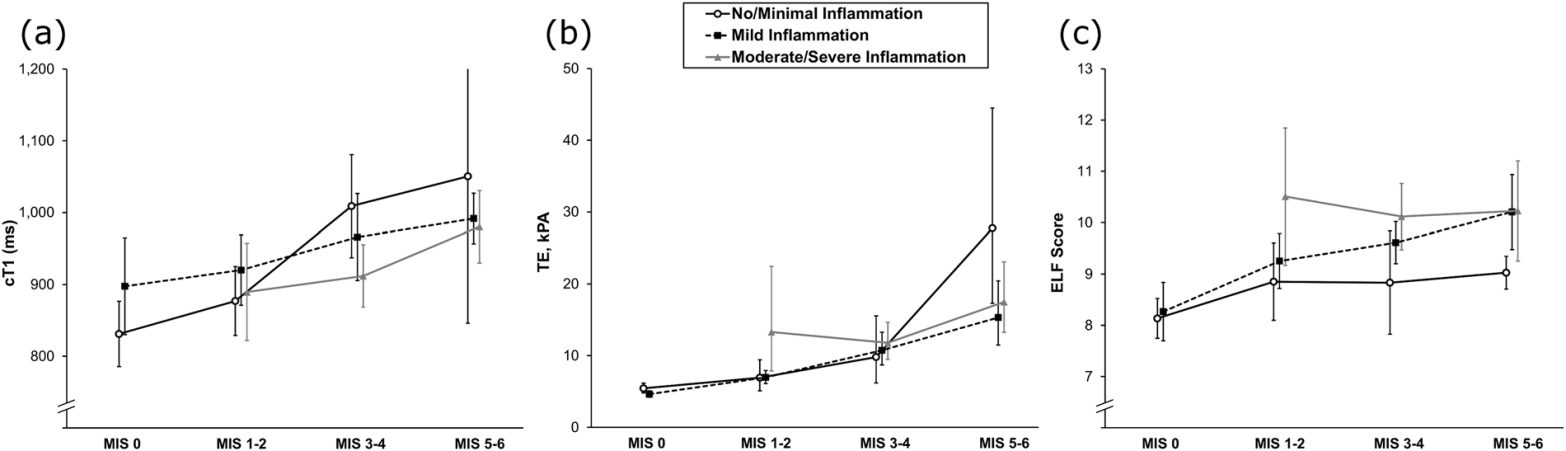
Multivariable analysis of inflammation and fibrosis. Plotted values are arithmetic means for cT1 and ELF score, and geometric means for TE, with the error bars representing 95% confidence intervals. Only one patient had no/minimal inflammation and a modified Ishak score (MIS) of 0, hence this point was excluded from the plots.

On multivariable analysis, after accounting for the effect of inflammation, ELF (*p* = 0·011), cT1 (*p* = 0·002) and TE (*p* < 0·001) remained significantly associated with fibrosis stage (Fig. 2). In addition, ELF was found to increase with the level of inflammation (*p* < 0.001), with values being 0·6 (SE = 0·3, *p* = 0·027) higher in patients with mild inflammation, and 1·2 (SE = 0·3, *p* < 0·001) higher in patients with moderate/severe inflammation, compared to those with no/minimal inflammation. No significant interaction effect between inflammation and fibrosis was detected for ELF (*p* = 0·641).

For TE, a significant interaction effect between inflammation and fibrosis was detected (*p* = 0·050). As shown in Fig. 2, the increase in TE with fibrosis was similar for patients in the no/minimal and mild inflammation groups. However, for patients with moderate/severe inflammation, the increase in TE by MIS was not observed, with a geometric mean TE of 13·3 (95% CI: 5·4–9·2) for MIS 1–2 and 17·5 (95% CI: 13·3–23·1) in MIS 5–6. A similar interaction effect was also observed in the analysis of cT1 (p = 0·050), with the magnitude of the increase with MIS becoming smaller with increasing levels of inflammation.

### Assessment of liver fat and iron by multiparametric MRI

There was a strong positive correlation between increasing histological fat (Brunt grade) and PDFF (r_s_ = 0·79, *p* < 0·001, n = 98) (Supplementary Fig. 2a). PDFF had excellent predictive accuracy for individual grades of steatosis, with AUROCs ranging from 0.90–0.94 (Supplementary Fig. 2a and Supplementary Table 4).

There was a negative correlation between liver iron content and T2* (r_s_ = −0·34, *p* < 0·001, n = 142), and a significant difference in T2* between patients with and without histological iron deposition (*p* < 0·001) (Supplementary Fig. 2b). In distinguishing patients with stainable iron from those without, T2* had an AUROC of 0·79 (95% CI 0·67–0·92, *p* < 0·001). At a cut-off of 18 ms, sensitivity was 83%, specificity 63%, PPV 25% and NPV 96%.

### Comparative performance of non-invasive tests to detect clinically significant liver disease

Median (IQR) cT1 and LIF score for the 22 healthy volunteers was 761 (741–811) ms and 0.76 (0.60–1.15) respectively, which was similar to the median cT1 and LIF score in patients with no evidence of liver disease on liver biopsy (cT1 787 (757–866) ms and LIF 0.91 (0.71–1.88), both *p* = 0.276). All non-invasive measurements were significantly different between patients with normal liver histology or simple steatosis and those with fibrosis and/or inflammation on biopsy (*p* < 0·0001 for all tests) (Table 4). To compare diagnostic accuracy between tests, a complete case analysis was performed on the subgroup of patients for whom adequate liver biopsy, cT1, TE and ELF measurements were all available. No significant difference was detected between the predictive accuracy of the three tests for the differentiation between normal biopsies/steatosis and the presence of any degree of inflammation and/or fibrosis (*p* = 0.500, n = 117, Supplementary Fig. 4 and Table 4). Following exclusion of post-liver transplant patients, cT1 showed superior predictive accuracy differentiating between these groups, although statistical significance was not achieved (*p* = 0.063, Table 4).

**Table 4:** Diagnostic accuracy of multiparametric MRI, ELF and TE in detecting clinically significant liver disease, with and without post-liver transplant patients included.

		Normal + simple steatosis	Inflammation and/or fibrosis	AUROC (95% CI)	*p*-value	Diagnostic test failure rate
All patients	cT1/LIF	23	119	0.76 (0.66–0.88)	** < 0.001**	4.3%
	ELF	23	124	0.80 (0.72–0.89)	** < 0.001**	1.9%
	TE	21	104	0.83 (0.74–0.91)	** < 0.001**	15%
Excluding post-transplant	cT1/LIF	13	89	0.89 (0.83–0.95)	** < 0.001**	6.1%
	ELF	19	94	0.77 (0.67–0.88)	** < 0.001**	1.7%
	TE	17	79	0.85 (0.76–0.93)	** < 0.001**	16.5%

### Comparative performance of non-invasive tests to detect significant liver fibrosis

Diagnosis of significant (moderate/severe) liver fibrosis identifies patients requiring close clinical follow up (including variceal and hepatocellular carcinoma surveillance in cirrhotics) and those most in need of therapeutic interventions to prevent progression of liver disease and/or decompensation, including participation in clinical trials. In order to compare the predictive accuracy of the tests in the context of clinical use, a set of net reclassification index (NRI) analyses were performed (Table 5). These compared the prognostic ability, with regards to significant liver fibrosis, between cT1, TE, ELF and the combination of TE and FIB-4, which was chosen as a ‘conventional diagnostic test’ that incorporates a non-patented serum score and TE. Based on the cut offs used, we found no significant difference between cT1 and either TE or ELF. The combination of TE/FIB-4 had poor predictive accuracy, with TE alone performing significantly better (*p* = 0.007).

**Table 5:** Net reclassification indices for the diagnosis of fibrosis stage≥3.

Tests		Total N	Reclassified Cases**	Cases Correctly Classified by		NRI	*p*-value
*Reference*	*Alternative*			*Reference Test*	*Alternative Test*		
ELF (>9.8)	cT1 (>875 ms)	140	63	29	34	0.08	0.615
TE (>13)	cT1 (>875 ms)	119	53	26	27	0.02	1.000
TE (>13)	ELF (>9.8)	123	25	16	9	−0.28	0.230
TE/FIB-4*	cT1 (>875 ms)	118	64	26	38	0.19	0.169
TE/FIB-4*	ELF (>9.8)	122	28	12	16	0.14	0.572
TE/FIB-4*	TE (>13)	124	15	2	13	0.73	**0.007**

^*^Cases with both TE > 13 and FIB-4 > 2.67 were treated as positive tests. **The number of cases classified differently by the two tests. *p*-values are from McNemar’s test, and bold values are significant at *p* < 0.05.

We performed a subgroup analysis that excluded post liver transplant patients (Supplementary Table 5), which found no significant difference between cT1 and either TE or ELF. As per the analysis of the whole cohort, predictive accuracy of the TE/FIB-4 combination was poor, and inferior to TE alone.

Subgroup analyses were also performed to assess the predictive accuracy of cT1 in those cases with indeterminate ELF and TE values. For those with indeterminate ELF (>7.7 and ≤9.8, n = 76), cT1 maintained good predictive accuracy for diagnosing significant fibrosis, as in the whole cohort (Table 6). However, cT1 was not found to be predictive of significant fibrosis in the n = 36 patients with borderline TE (>7 and ≤13).

**Table 6:** Diagnostic accuracy of cT1 in detecting fibrosis stage ≥3 in cases with borderline ELF and TE.

Inclusion Criteria	N	AUROC for cT1 (95% CI)	p-Value	Se	Sp	PPV	NPV
ELF > 7.7 and ≤9.8	76	0.70 (0.58–0.82)	**0.003**	84	55	57	83
TE > 7 and ≤13	36	0.55 (0.33–0.76)	0.657	96	31	71	80

Se, sensitivity; Sp, specificity; NPV, negative predictive value; PPV, positive predictive value. Bold *p*-values are significant at *p* < 0.05.

## Discussion

Validated non-invasive tools for the diagnosis, stratification and monitoring of liver disease are an urgent requirement to streamline clinical care pathways and facilitate drug development. One of the emerging imaging-based technologies is LMS, a rapid non-contrast multiparametric MRI scan quantifying hepatic fibro-inflammatory injury, fat and iron. Here we describe the first independent validation study of LMS where, critically, its performance was also evaluated alongside other commonly used non-invasive biomarkers.

MRI scanners are available in most UK hospitals and, in contrast to MRE, LMS can be implemented on any modern clinical 1.5 or 3.0 tesla MR scanner with no additional hardware requirements. MRI with LMS is not contraindicated in patients with metal implants, as any modern (within the last 20 years) implants are almost 100% non-ferrous.

Crucially for an emerging diagnostic test, we showed that MRI with LMS had excellent repeatability and reproducibility (immediate test-retest and repeated over 10 weeks). Additionally, unlike other non-invasive imaging tests, quantification of fibro-inflammatory injury was unaffected by post-prandial state, potentially increasing ease of use and patient acceptability by avoiding pre-scan fasting. Immediate test-retest repeatability of LMS has also been confirmed in a mixed healthy volunteer/patient cohort (unpublished data).

We have validated, in a diverse unselected secondary care population, that LMS has good diagnostic accuracy for detecting fibro-inflammatory injury, fat and iron in the liver across a range of disease severity and aetiology when compared to the current imperfect ‘gold standard’ of liver biopsy. We acknowledge the unavoidable patient selection bias introduced by the requirement for liver biopsy, as well as the need for further studies to define applicability of LMS in other patient groups.

Based on the ROC analysis (Table 3), TE was superior to both multiparametric MRI and ELF test in the detection of moderate and severe fibrosis, whilst all three non-invasive tests were comparable for the detection of any fibrosis. Both for TE and LMS, a significant interaction effect between inflammation and fibrosis was detected. As T1 relaxation time is influenced by the presence of inflammation^27^ and liver fat^28^, a more nuanced understanding of the individual and combined effects of fibrosis, inflammation, fat and iron on T1 relaxation time in different aetiologies of liver disease is required to refine the interpretation of LMS. However, the multiparametric MRI sequence is ideally suited to correct for these distinct histological variables.

LMS, ELF and TE had comparable predictive accuracy for the differentiation between normal biopsies/steatosis and the presence of any degree of inflammation and/or fibrosis. On a post hoc analysis, we have identified that liver transplantation may influence the diagnostic performance of cT1 in detecting significant liver disease, with a substantial but not significant improvement in diagnostic accuracy observed (AUROC 0.89 vs. 0.76) when post-transplant patients were excluded. To investigate this further, a prospective multicentre trial comparing the accuracy of LMS against a liver biopsy in the assessment of liver transplant recipients is ongoing (NCT03165201).

Following net reclassification analysis, cT1 was comparable to TE and ELF in diagnosing significant liver fibrosis, when using the proposed cut off of 875 ms. Moreover, our analysis suggested that cT1 could be used to increase the diagnostic yield in indeterminate ELF cases, although this was not true for indeterminate TE cases. These findings have particular relevance in NAFLD patients, in whom National Institute for Health and Care Excellence (NICE) guidance recommends the ELF test for the identification of liver fibrosis^29^, and where TE failure rates are highest due to obesity, currently necessitating further evaluation via alternative imaging modalities such as acoustic radiation force impulse (ARFI) imaging, shear wave elastography or by liver biopsy. A recent decision analytic model for NHS patients with suspected NAFLD suggested that inclusion of LMS either as an adjunct to or replacement for TE in clinical care pathways may lead to cost savings by reducing the number of liver biopsies^30^.

LMS demonstrated 98.1% technical success rate even in obese subjects, which compares favourably with the average TE success rate of 85%, even with the XL probe. The high technical success rate of LMS has been corroborated in 5000 subjects included in the UK Biobank cohort (96.4% for PDFF estimation)^31^.

In contrast to the previous study by Banerjee *et al*.^17^, we used the well-established modified Dixon method for PDFF to quantify liver fat, a technique that is widely applicable across different MRI platforms and is highly correlated with MR spectroscopy and also hepatic triglyceride levels^15^. Additionally, MRI-PDFF is more reliable than Fibroscan Controlled Attenuation Parameter in obese individuals^32^. We have recently reported a subgroup analysis of multiparametric MRI in NAFLD patients from our study cohort^33^.

LMS also incorporates gold standard MRI assessment of liver iron^34,35^ performed with a high degree of accuracy (sensitivity and NPV of 83% and 96% respectively), with potential application in the diagnosis and monitoring of hereditary and acquired conditions leading to iron overload.

The optimal LMS cut-off points for stratification of different CLD stages will require further investigation in different research settings and may be disease-specific. However, based on the application of LMS MRI in the UK Biobank cohort, a LIF score of ≤0.82 (95% CI 0.72–0.95) has been validated as a normal value for the UK population. Thus, in a general population setting, a LIF score of ≤1 with its high sensitivity (86%) would strongly indicate the absence of significant fibro-inflammatory liver disease, allowing identification of low-risk patients who could be spared further investigations and unnecessary follow-up. In contrast, a LIF cut-off >2 identified CLD patients with an increased risk of clinical outcomes^18^ and could therefore potentially be used to enrich trial populations prior to enrolment in interventional studies, or as a clinically meaningful surrogate endpoint. Given that emerging treatments (and potential combination regimens) for CLD may modulate fat and fibro-inflammatory components of liver injury to a variable extent, quantitative multiparametric MRI assessments may be well suited for drug development, especially in NASH.

In this first “in-the-field” study, LMS demonstrated good diagnostic accuracy for detecting fibro-inflammatory injury, fat and iron in the liver across a range of disease severity and aetiology and with excellent repeatability and reproducibility. Its diagnostic performance was comparable to existing well-validated non-invasive biomarkers. Overall, our findings support the further development and refinement of multiparametric MRI technology for assessment of liver disease.

## Electronic supplementary material

Supplementary Information
