# Supplementary material for: Multiparametric magnetic resonance imaging for quantitation of liver disease: a two-centre cross-sectional observational study
Source: Sci Rep. 2018 Jun 15;8:9189. doi: 10.1038/s41598-018-27560-5 (PMC6003924; doi:10.1038/s41598-018-27560-5)
Supplement: Supplementary file 1 — Supplementary Information [file 41598_2018_27560_MOESM1_ESM.pdf]

Multiparametric magnetic resonance imaging for quantitation of liver disease: a two-centre cross-sectional observational study

Natasha McDonald[1*], Peter J. Eddowes[2*], James Hodson[3], Scott I.K. Semple[4], Nigel P. Davies[5], Catherine J. Kelly[6], Stella Kin[6], Miranda Phillips[6], Amy H. Herlihy[6], Timothy J. Kendall[1,7], Rachel M. Brown[8], Desley A.H. Neil[8], Stefan G. Hübscher[2,8], Gideon M. Hirschfield[2†], Jonathan A. Fallowfield[1†]

[*] These authors contributed equally to this work.

[†] These authors share senior authorship.

Affiliations:

1. MRC/University of Edinburgh Centre for Inflammation Research, Queen’s Medical Research Institute, Edinburgh, EH16 4TJ, UK
2. Centre for Liver Research and NIHR Birmingham BRC, University of Birmingham, Birmingham, B15 2TT, UK
3. Institute of Translational Medicine, University Hospitals Birmingham NHS Foundation Trust, Birmingham, B15 2TT, UK
4. BHF/ University of Edinburgh Centre for Cardiovascular Science, Queen's Medical Research Institute, Edinburgh, EH16 4TJ, UK
5. Imaging and Medical Physics, University Hospitals Birmingham NHS Foundation Trust, Birmingham, B15 2TH, UK

6. Perspectum Diagnostics Ltd., Oxford Centre for Innovation, Oxford, OX1 1BY, UK
7. Division of Pathology, Royal Infirmary of Edinburgh, Edinburgh, EH16 4SA, UK
8. Department of Cellular Pathology, Queen Elizabeth Hospital, Birmingham, B15 2TH, UK

Corresponding Author: Dr Jonathan A. Fallowfield, MRC/ University of Edinburgh Centre for Inflammation Research, Queen's Medical Research Institute, Edinburgh, EH16 4TJ, UK. Tel: +44(0)131-242-6655. Email: Jonathan.Fallowfield@ed.ac.uk. Orcid ID: orcid.org/0000-0002-5741-1471.

SUPPLEMENTARY MATERIAL – CONTENTS

**Supplementary Methods**	Pages 4-5
**Supplementary Figures**	Pages 6-9
**Supplementary Tables**	Page 10-12
**STARD Checklist**	Page 13

SUPPLEMENTARY METHODS

Magnetic resonance imaging and image analysis

MRI data were acquired using a combination of body matrix and spine matrix coil elements positioned for optimal coverage of the liver. Transverse abdominal T1 and T2* MR maps were acquired for the estimation of extracellular fluid and liver iron respectively. Liver fat, as quantified by the proton density fat fraction (PDFF, %) was measured by a modified Dixon method. Briefly, a fast gradient echo sequence was performed to acquire in-phase and out-of-phase images with the following parameters: repetition time 500ms; echo times 1.23, 2.46, 3.69, 4.92, 6.15, 7.38, 8.61, and 9.84ms; flip angle of 6 and 20 degrees; section thickness of 6mm.
As the presence of iron overload is known to shorten the T1 relaxation time, a proprietary algorithm was applied to calculate the iron-corrected T1 (cT1).[17] To facilitate interpretation, the clinically-relevant range of liver cT1 values was mapped onto a simple 0 to 4 scale called the Liver Inflammation and Fibrosis (LIF) Score. The mapping is monotonic and trilinear: cT1 values between 650ms and 800ms are linearly-mapped to the LIF interval 0 to 1; cT1 values between 800ms and 950ms are linearly-mapped to the LIF interval 1 to 3; cT1 values between 950ms and 1300ms are linearly-mapped to the LIF interval 3 to 4. cT1 values less than 650ms or greater than 1300ms map to a LIF score of 0 or 4, respectively.

The LMS software platform was used to analyse anonymised images off-site to generate cT1, LIF, T2* and PDFF values in a single operator-defined region of interest in the right liver lobe, away from vascular and biliary structures.

Reproducibility and repeatability of multiparametric MRI

To assess the inter-test reproducibility of multiparametric MRI, ten healthy volunteers were scanned, removed from the scanner and immediately re-scanned. The effect of dietary intake on cT1 and T2* was investigated in 15 healthy volunteers by performing an initial scan after an overnight fast, followed by unrestricted non-standardised dietary intake and a repeat scan within 90 minutes of food ingestion. To investigate intra-subject repeatability over time, five healthy volunteers were scanned once a week for a period of 9-10 weeks. There was no change in the subjects' medical condition or medication history over the study period. For all the healthy volunteer scans, the scanning protocol and data analysis were identical to that performed on patients. For PDFF measurements, values of <2% were excluded as they lie below the minimal threshold of accurate analysis by MRI.

SUPPLEMENTARY FIGURES

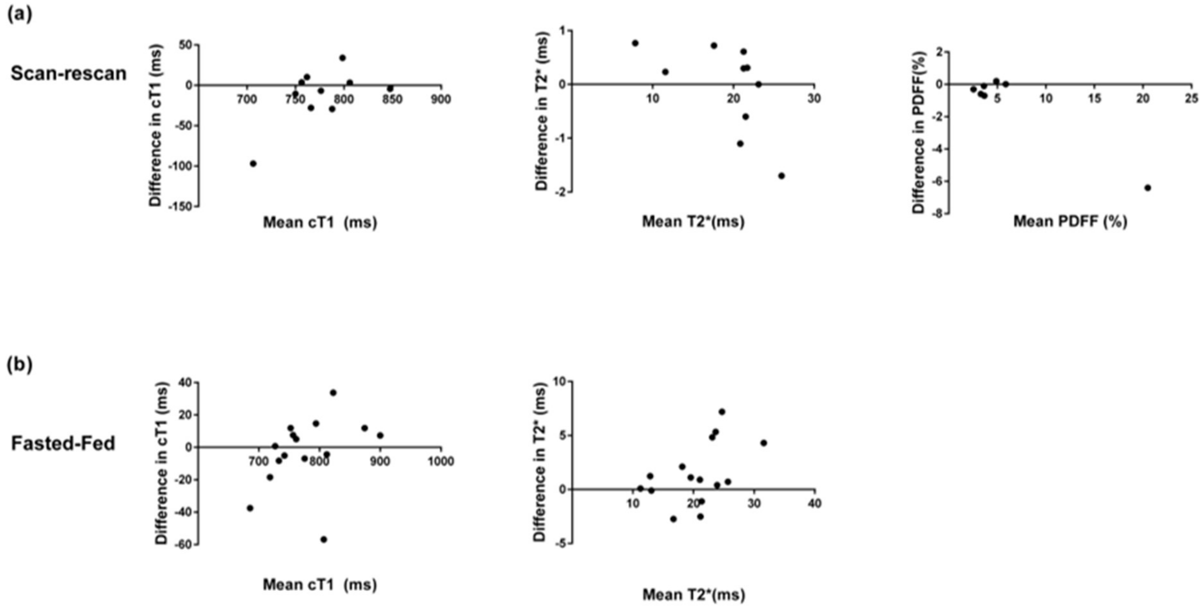

Supplementary Fig. 1. Bland-Altman plots of differences in cT1, T2*, and PDFF against the mean of the two measurements. (A) Scan and immediate re-scan. (B) Scanned fasted and re-scanned fed.

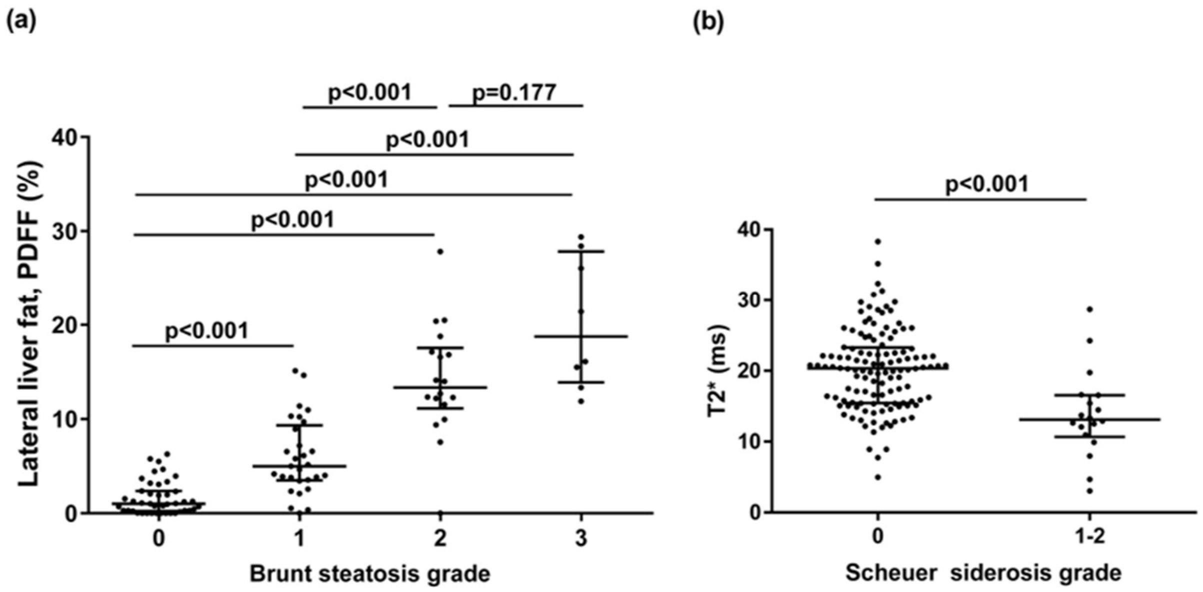

Supplementary Fig. 2. **Steatosis and siderosis assessment.** There was a significant stepwise increase in PDFF (A) with increasing grade of steatosis (Jonckheere-Terpstra test, *p*<0·001). P-values are for the post-hoc pairwise comparisons between steatosis grades. (B) Reduction in T2* with increasing Scheuer siderosis grade (Mann-Whitney test; *p*<0·001). The line and whiskers represent median and interquartile range.

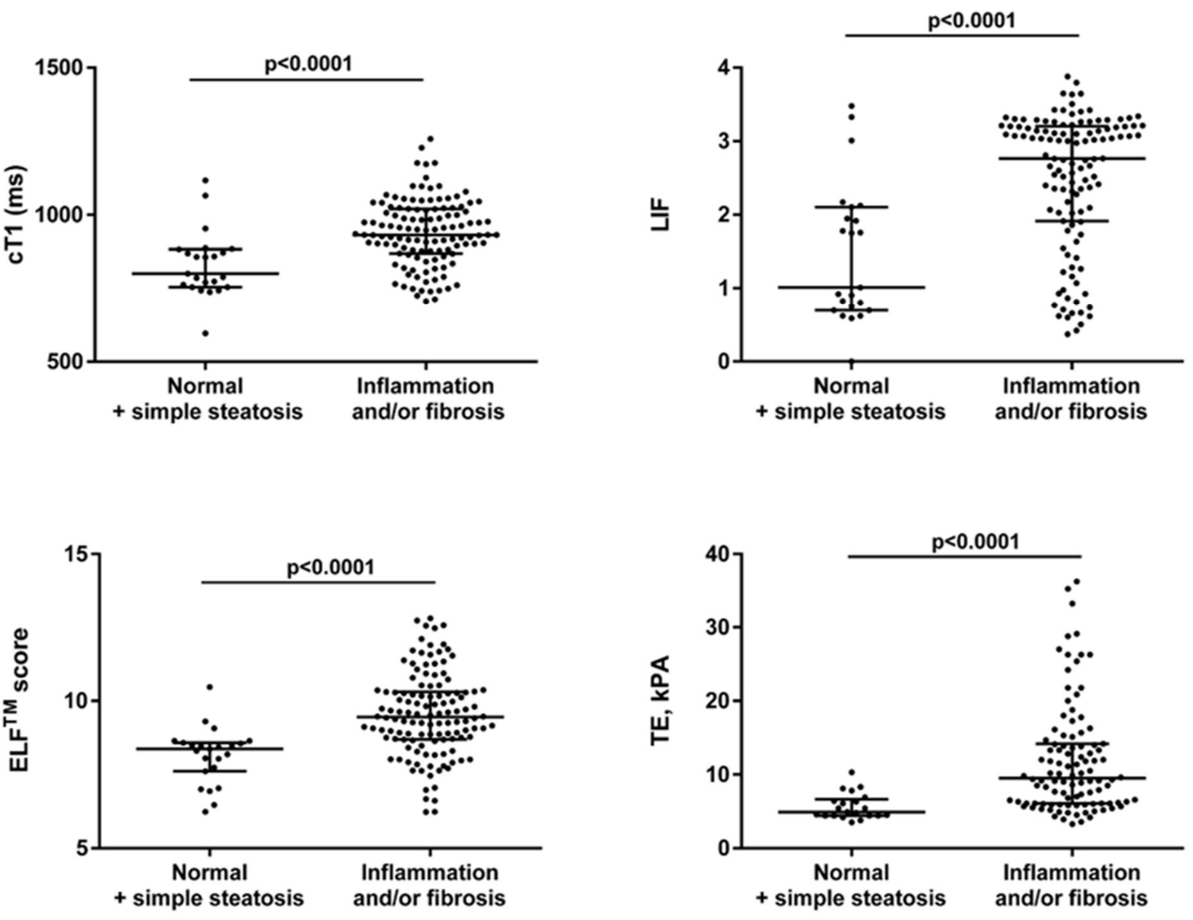

Supplementary Fig. 3. Multiparametric MRI, ELF and TE in assessing clinically significant liver disease (normal/simple steatosis versus any degree of inflammation/fibrosis). Mann-Whitney test, p<0·0001 for all. The line and whiskers represent median and interquartile range.

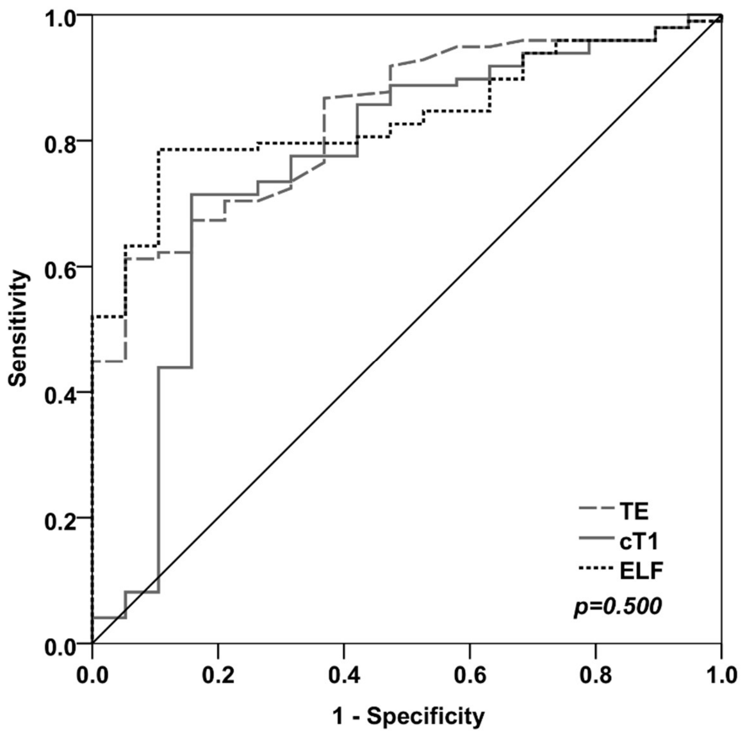

Supplementary Fig. 4. ROC curve for the differentiation of patients with normal biopsies/simple steatosis from patients with any degree of inflammation/fibrosis. This is a complete cases analysis, including only those cases with adequate liver biopsy, cT1, TE and ELF measurements, and is based on n=117. No significant differences were detected between the three AUROCs (*p*=0·500).

SUPPLEMENTARY TABLES

Score	Description	Fibrosis Severity
0	No fibrosis	None
1	Zonal fibrosis involving a minority of portal tracts and/or zone 3 areas	Mild
2	Zonal fibrosis involving a majority of portal tracts and/or zone 3 areas	
3	Bridging fibrosis - occasional foci	Moderate
4	Bridging fibrosis – widespread	
5	Bridging fibrosis – widespread, with occasional nodules (incomplete/early cirrhosis)	Severe
6	Cirrhosis, probable or definite	

Supplementary Table 1. Modified Ishak Score (MIS).

	Fibrosis Observer 1							Fat Observer 1				Iron Observer 1		
**Observer 2**	**0**	**1**	**2**	**3**	**4**	**5**	**6**	**0**	**1**	**2**	**3**	**0**	**1**	**2**
**0**	5	1	0	1	0	0	0	10	5	0	0	34	2	1
**1**	7	4	2	0	0	0	0	3	17	0	0	5	3	0
**2**	0	3	4	1	0	0	0	0	3	2	0	0	0	0
**3**	2	5	1	1	0	0	0	0	0	2	3			
**4**	0	0	3	0	1	0	0							
**5**	0	0	1	0	0	0	1							
**6**	0	0	0	0	0	0	2							

Supplementary Table 2. Interobserver agreement for histological assessment of liver fibrosis, fat and iron.

	Multiparametric MRI (n=108)							ELF test (n=113)						TE (n=96)	
**Fibrosis stage (MIS)**	**AUROC (95% CI)**	**Cut off levels cT1 (ms)**	**LIF**	**Se**	**Sp**	**PPV**	**NPV**	**AUROC (95% CI)**	**Cut off levels**	**Se**	**Sp**	**PPV**	**NPV**	**AUROC (95% CI)**	***p*-value***
≥1	0.82 (0.71 – 0.92)	800	1	91	48	87	58	0.76 (0.66 – 0.87)	7·7	92	22	82	42	0.83 (0.73 – 0.93)	0.329
≥3	0.74 (0.65 – 0.84)	875	2	91	56	67	86	0.76 (0.67 – 0.84)	9·8	46	86	76	62	0.86 (0.78 – 0.94)	0.051
≥5	0.74 (0.64 – 0.83)	950	3	77	60	33	91	0.73 (0.62 – 0.84)	11·3	17	97	57	81	0.88 (0.82 – 0.95)	**0.003**

Supplementary Table 3. Diagnostic accuracy of multiparametric MRI, ELF and TE in detecting any, moderate-to-severe and severe liver fibrosis in non-transplant patients (n=115). Se, sensitivity; Sp, specificity; NPV, negative predictive value; PPV, positive predictive value. All AUROCs were significant at *p*<0·001. **p*-

values comparing the AUROCs of multiparametric MRI vs. ELF vs. TE for the n=88 patients with data available for each measure. Significant comparisons (bold) were followed by post-hoc pairwise comparisons, the *p*-values of which were Bonferroni corrected to account for multiple comparisons. These comparisons found TE to be significantly superior to both multiparametric MRI and ELF test in the detection of MIS ≥5 (*p*=0.002, 0.029). ** TE cut off levels not included as they are specific to aetiology of liver disease.

**Brunt steatosis grade**	**Multiparametric MRI PDFF (%) (n=98)**					
	**AUROC (95% CI)**	**Cut off levels**	**Se**	**Sp**	**PPV**	**NPV**
≥1	0·90 (0·84-0·97)	6.4	65	100	100	69
≥2	0·94 (0·87-1·00)	10	85	92	79	94
≥3	0·94 (0·89-0.99)	22.1	38	99	75	95

Supplementary Table 4. Diagnostic accuracy of multiparametric MRI (PDFF) in detecting hepatic steatosis grades. Se, sensitivity; Sp, specificity; NPV, negative predictive value; PPV, positive predictive value. All AUROCs were significant at $p<0{\cdot}0001$.

**Tests**		**Total**	**Reclassified**	**Cases Correctly Classified by**			***p*-**
Reference	***Alternative***	**n**	**Cases****	***Reference Test***	***Alternative Test***	**NRI**	**value**
ELF (>9.8)	cT1 (>875ms)	106	47	20	27	0.15	0.382
TE (>13)	cT1 (>875ms)	90	39	15	24	0.23	0.200
TE (>13)	ELF (>9.8)	94	16	8	8	0.00	1.000
TE/FIB-4*	cT1 (>875ms)	89	49	16	33	0.35	**0.021**
TE/FIB-4*	ELF (>9.8)	93	20	6	14	0.40	0.115
TE/FIB-4*	TE (>13)	95	12	2	10	0.67	**0.039**

Supplementary Table 5. Net reclassification indices for the diagnosis of fibrosis stage ≥3 (excluding liver transplant recipients). *Cases with both TE>13 and FIB-4>2.67 were treated as positive tests. **The number of cases classified

differently by the two tests. *p*-values are from McNemar’s test, and bold values are significant at $p<0.05$.

Standards for Reporting Diagnostic accuracy studies (STARD) 2015 Checklist

Section & Topic	No	Item	Reported on page #
**TITLE OR ABSTRACT**			
	**1**	Identification as a study of diagnostic accuracy using at least one measure of accuracy<br>(such as sensitivity, specificity, predictive values, or AUC)	3
**ABSTRACT**			
	**2**	Structured summary of study design, methods, results, and conclusions<br>(for specific guidance, see STARD for Abstracts)	3
**INTRODUCTION**			
	**3**	Scientific and clinical background, including the intended use and clinical role of the index test	4, 5, 6
	**4**	Study objectives and hypotheses	6
**METHODS**			
Study design	**5**	Whether data collection was planned before the index test and reference standard<br>were performed (prospective study) or after (retrospective study)	7
Participants	**6**	Eligibility criteria	7
	**7**	On what basis potentially eligible participants were identified<br>(such as symptoms, results from previous tests, inclusion in registry)	7
	**8**	Where and when potentially eligible participants were identified (setting, location and dates)	7
	**9**	Whether participants formed a consecutive, random or convenience series	7
Test methods	**10a**	Index test, in sufficient detail to allow replication	8, 9, Suppl methods
	**10b**	Reference standard, in sufficient detail to allow replication	8
	**11**	Rationale for choosing the reference standard (if alternatives exist)	No alternatives
	**12a**	Definition of and rationale for test positivity cut-offs or result categories<br>of the index test, distinguishing pre-specified from exploratory	15
	**12b**	Definition of and rationale for test positivity cut-offs or result categories<br>of the reference standard, distinguishing pre-specified from exploratory	9, 10, 15, 19
	**13a**	Whether clinical information and reference standard results were available<br>to the performers/readers of the index test	8
	**13b**	Whether clinical information and index test results were available<br>to the assessors of the reference standard	8
Analysis	**14**	Methods for estimating or comparing measures of diagnostic accuracy	10, 11
	**15**	How indeterminate index test or reference standard results were handled	n/a
	**16**	How missing data on the index test and reference standard were handled	7, 8
	**17**	Any analyses of variability in diagnostic accuracy, distinguishing pre-specified from exploratory	n/a
	**18**	Intended sample size and how it was determined	9, 10
**RESULTS**			
Participants	**19**	Flow of participants, using a diagram	12 (Fig 1)
	**20**	Baseline demographic and clinical characteristics of participants	13
	**21a**	Distribution of severity of disease in those with the target condition	13
	**21b**	Distribution of alternative diagnoses in those without the target condition	n/a
	**22**	Time interval and any clinical interventions between index test and reference standard	7
Test results	**23**	Cross tabulation of the index test results (or their distribution)<br>by the results of the reference standard	15, 17, 18

	**24**	Estimates of diagnostic accuracy and their precision (such as 95% confidence intervals)	15, 17, 18, 19, 20
	**25**	Any adverse events from performing the index test or the reference standard	n/a
**DISCUSSION**			
	**26**	Study limitations, including sources of potential bias, statistical uncertainty, and generalisability	23, 24
	**27**	Implications for practice, including the intended use and clinical role of the index test	23, 24
**OTHER INFORMATION**			
	**28**	Registration number and name of registry	7
	**29**	Where the full study protocol can be accessed	Suppl material file
	**30**	Sources of funding and other support; role of funders	27, 28
